# Prognostic value of epigenetic markers for canine mast cell cancer

**DOI:** 10.1371/journal.pone.0283616

**Published:** 2023-03-30

**Authors:** Shahzar Syed, Britta J. Knight, Geoffrey A. Wood, Brenda Lynn Coomber

**Affiliations:** 1 Department of Biomedical Sciences, Ontario Veterinary College, University of Guelph, Guelph, Ontario, Canada; 2 Department of Pathobiology, Ontario Veterinary College, University of Guelph, Guelph, Ontario, Canada; Colorado State University, UNITED STATES

## Abstract

Canine Mast cell tumors (MCTs) constitute approximately 21% of all canine skin tumors. Despite the use of comprehensive grading systems, biological aggressiveness is sometimes difficult to predict, therefore there is a need for better prognostic markers. Progression in various cancers involves DNA hypermethylation, hypomethylation and epigenetic enzyme dysregulation. Therefore, global levels of 5-methylcytosine, 5-hydroxymethylcytosine and associated enzymes DNMT1, and IDH1 expression may predict MCT aggressiveness. A tissue microarray (TMA) with cores from 244 different tumor samples from 189 dogs was immunolabelled and used to quantify the global DNA methylation and hydroxymethylation levels as well as the levels of the enzymes involved in DNA methylation and their relationship with canine MCT outcome. From the immunolabelled TMA, H-scores were generated using QuPath (v0.1.2) and analyzed with associated patient data. High 5MC and DNMT1, and low IDH1 levels were associated with poorer outcome when looking at all canine MCT cases. High 5MC levels showed significance for shorter disease-free interval (DFI) in subcutaneous cases and high 5MC levels showed poorer DFI and overall survival (OS) in cases with Kiupel’s grading system high grade. Cases with grade II in Patnaik’s grading system showed better DFI with low levels of DNMT1 and better OS with low levels of 5MC and 5HMC. High levels of DNMT1 staining were also associated with shorter DFI for dermal MCTs. For cases that received adjuvant therapy in addition to surgery, all parameters except IDH1 were significantly associated with OS. Therefore, there is potential for DNA methylation status and levels of enzymes associated with DNA methylation pathways to better predict outcome in canine MCT, and to possibly influence treatment decisions.

## Introduction

Epigenetic events allow for an adjustment in gene function and activity without permanent alterations of DNA sequence [1]. Modification of DNA methylation is an epigenetic event where a methyl group is covalently added to the 5’ carbon of the cytosine base on its pyrimidine ring. This activity is performed by either the maintenance DNA methyltransferase enzyme DNMT1 (which re-establishes methylation patterns after DNA replication or repair), or by the de novo group of DNA methyltransferase enzymes DNMT3a and 3b (which establish new methylation patterns) [2]. Methylation of cytosine is an epigenetic event often dysregulated in cancers, especially in hematopoietic malignancies of the myeloid cell lineage such as acute myeloid leukemia (AML) and myelodysplastic syndrome (MDS) [3].

Active DNA demethylation is initiated by the oxidation of 5-methyl-cytosine (5mC) into 5-hydroxymethylcytosine by the Ten-Eleven Translocation (TET) protein family enzymes TET1, 2 and 3 [4, 5]. TET2 is frequently dysregulated and mutated, especially in haematological malignancies and malignancies of myeloid cells [6, 7]. Mast cell progenitor cells from TET2 deficient mice showed reduced differentiation into mature cells [8], and loss of TET2 led to impaired differentiation of mature mast cells from bone marrow mast cell progenitors [9]. Isocitrate dehydrogenase (IDH) 1 and 2 are metabolic enzymes that function in the tricarboxylic acid cycle to catalyze the oxidative decarboxylation of isocitrate to α-ketoglutarate in the cytosol and the mitochondria, respectively [6]. IDH mutations are seen frequently in myeloid malignancies, including AML (Acute Myeloid Leukemia) [10]. IDH1 and IDH2 mutations often lead to a gain of function and eventual reduction in TET enzyme activity [6, 10, 11]. Hence, dysregulation of DNA methylation may be important in canine mast cell cancer progression.

Mast cells are heterogeneous immune system cells that differentiate and mature in tissue-specific ways once they have migrated to their respective sites [12]. These cells are prone to malignant transformation in domestic dogs. Mast cell tumors (MCTs) are a prevalent neoplasm in dogs, and account for up to 21% of all skin tumors. Dogs that present with MCT are generally between the age of 7.5 and 9 years, with most of these neoplasms occurring in the dermis and subcutaneous tissue [13, 14]. There is a wide variability in the biological behavior of canine MCT ranging from benign, which are easily treated and do not recur, to highly aggressive and metastatic, which have poor outcomes [15]. Although the majority of MCTs can be successfully treated, aggressive and metastatic MCTs have a poor prognosis—yet it can be challenging to differentiate them from the less aggressive MCTs [16]. While accurate prognostication of many canine MCTs is achieved by the current grading schemes and molecular markers, there are a significant subset of MCTs whose biological behaviour remains difficult to predict [15, 17–20]. Therefore, it is important to identify additional prognostic markers for both cutaneous/dermal and subcutaneous MCTs. Epigenetic DNA methylation marks, as well as the enzymes involved in cytosine methylation and demethylation are often dysregulated in various cancers. Since epigenetic regulation is important in the development and disease progression of myeloid cells including mast cells, dysregulation of epigenetic markers and enzymes could be useful in determining more aggressive canine MCTs. This study was conducted to determine if the levels of 5MC, 5HMC, DNMT1, or IDH1 are prognostic in canine MCT.

## Materials and methods

### SDS-PAGE and western blotting

Protein lysates were generated from canine mast cell lines MCT1 (adherent cell line) and MCT2 (suspension cell line) [21], canine B-cell lymphoma cell line CBCL-1 (a gift from Dr. Barbara Rütgen, University of Veterinary Medicine, Vienna, Austria) [22], and canine kidney MDCK cell line (ATCC, CCL-34) [23]. Human Liver Whole Tissue Lysate (Adult Whole Normal) NB820-59234 was purchased from Novus Biologicals. Antibodies used for detecting proteins of interest were: DNMT1 (Abcam anti-DNMT1 antibody [EPR18453] ab188453), IDH1 (Abcam anti-IDH1 antibody [EPR12296] ab172964).

SDS-PAGE and western blotting was performed as previously described [21] using a 10% resolving gel, 30 μg of total protein from cell line lysates, and 10 μg total protein for Human Liver Whole Tissue Lysate. Proteins were transferred to polyvinylidene difluoride (PVDF) membrane (Millipore Sigma) by bulk wet transfer, then membranes were washed, blocked for one hour in buffer containing 5% skim milk powder, then incubated with primary antibody (1:1000 dilution) with rocking overnight at 4°C. After washing, membranes were incubated with rocking for 1 hour at room temperature with 1:10,000 dilution of goat anti-rabbit secondary antibody (Millipore Sigma) in blocking solution. Membranes were then washed and bands detected with Immobilon Forte (Millipore Sigma) in a BioRad ChemiDoc XRS+ instrument. See S1 Fig for raw blot images.

### Immunohistochemistry

All tissues used in this study were from veterinary patients undergoing surgical procedures for the detection and treatment of their disease. Accordingly, under the regulations of the Canadian Council on Animal Care, as implemented by the University of Guelph Animal Care Committee, ethical approval for animal use was not required. To quantify tissue immunolabelling for 5MC, 5HMC, DNMT1, and IDH1, a previously generated tissue microarray (TMA) using standard immunohistochemistry techniques was employed [24]. In addition to those for western blot, we also used antibodies to 5MC (Millipore Sigma Anti-5-methylcytosine, clone 33D3) and 5HMC (Invitrogen 5-Hydroxymethylcytosine Monoclonal Antibody [RM236]). The TMA contained 244 tumour samples from 189 dogs, distributed with control tissues over two blocks. Clinical outcome data were associated with 115 of the cases. Antigen retrieval was achieved using a solution of 10 mM sodium citrate buffer, pH 6.0, for all markers except for 5HMC, which used EDTA pH 9.0. For 5MC and 5HMC detection only, the slides were incubated for 15 minutes in 3.5M HCl to denature DNA prior to blocking. Primary antibody dilutions were: 5MC 1 μg/mL, 5HMC 1:2000, DNMT1 1:100, IDH1 1:100; all were incubated overnight at 4°C. For negative controls, primary antibodies were omitted and slides underwent incubation under equivalent conditions (see S2 Fig). Specificity was also evaluated with isotype control antibodies, as previously described [24]. All grading and optimization of immunodetection was performed on full face sections of canine MCT specimens. The HRP labelled systems were applied to the slides and incubated for 30 minutes at room temperature. Immunolabelling for 5MC and 5HMC utilized the Dako EnVision HRP labelled Dual Link System, and for DNMT1 and IDH1 utilized the Dako Labelled Polymer-HRP Anti-Rabbit system. All Dako labelling systems were employed as per manufacturer’s protocols. Slides were then rinsed twice in Tris Buffer for 5 minutes, and Abcam DAB substrate Kit was used for immunodetection according to manufacturer’s instructions. DAB incubation times were 10 minutes for 5MC and 5HMC, 15 minutes for DNMT1 detection, and 6 minutes for IDH1 detection. Slides were counterstained with Modified Harris hematoxylin (Fisher, SH26), and coverslipped with Cytoseal XYL (Thermo Fisher).

### Immunohistochemistry analysis

Slides were scanned by the Ontario Institute for Cancer Research (OICR) on an Aperio ScanScope, and files were generated in STS format for analysis of immunolabelling on QuPath (0.1.2) open-sourced quantitative pathology software [25]. Aided by the workflow macro included in the software, TMAs underwent colour deconvolution based on the DAB staining and hematoxylin detection system, separating the staining into layers which could be analyzed independently to accurately determine immunolabelling of each cell. The TMA was then de-arrayed and individual cores identified and mapped to relate immunolabelling data generated from QuPath with the clinical data associated with each core. QuPath was trained to identify canine mast cell tumour cells (see S3 Fig), and values ranging from 0 pixel intensity (for no detectable DAB reaction product), 1 for low pixel intensity, 2 for moderate pixel intensity, and 3 for high pixel intensity staining were assigned to each cell for each immunolabelling target. These were then multiplied by the percentage of total cells stained for each value to generate an H-Score for each core. The cores were then individually examined for quality control, and regions of cores that had collagen or presence of edema or other structures that lead to aberrant cell identification were eliminated by manual annotation. Cores with less than 30% of the tissue having neoplastic cell representation or cores that were lost during IHC and sectioning were eliminated from analysis. An average H-score was calculated for cases with multiple cores/TMA. H-scores were then linked to the clinical data associated with the cases and results were analyzed for outcome.

### Statistical analysis

The X-tile program [26] was used to determine statistically significant cut-points for survival analysis using visual representation of H-scores in a histogram to stratify the H-scores into statistically significant high and low groups. Using these X-tile cut points, Kaplan-Meier curves were generated, and log-rank analysis was performed for disease free interval (DFI) and overall survival (OS) using GraphPad Prism 9. DFI was defined as the time from the date of surgical removal to local recurrence or metastasis of the MCT. Dogs not progressed at last follow up, or lost to follow up, were right censored for DFI analysis. OS was defined as the time from the date of surgical removal to the time of euthanasia or death due to causes related to MCT. Dogs alive at last follow up, or that died or were euthanized for reasons unrelated to MCT were right censored for OS analysis. Cases stratified by location (dermal vs subcutaneous MCT) were analyzed for DFI and cases assessed as high grade using Kiupel’s grading system, or grade II using Patnaik’s grading system were also analyzed for DFI; there were insufficient events in these groups when stratified for OS to perform meaningful outcome analysis. Details of specimen assessment for histopathological grade are found in our previous publication [24]. A value of *p* < 0.05 was considered statistically significant for all analyses.

## Results

**Fig 1:**
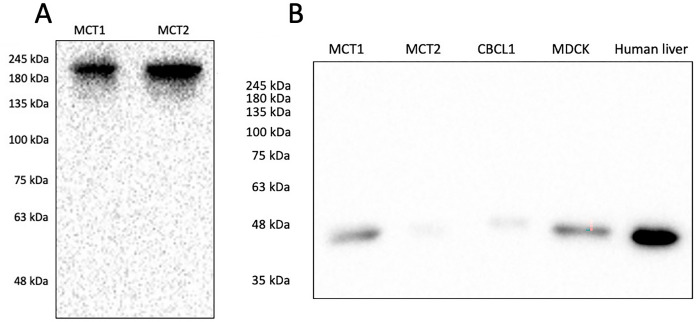
Western blots showing validation of antibodies used for immunohistochemistry. **A**) DNMT1: predicted molecular mass 183 kDa; **B**) IDH1: predicted molecular mass 47 kDa. MCT1, MCT2: canine mast cell cancer cell line lysates; CLBL1: canine B-cell lymphoma cell line lysate; MDCK: Madin-Darby canine kidney cell line lysate; Human liver: commercially available lysate for western blotting.

Antibodies specifically raised against canine protein targets used in this study were not commercially available. Therefore, western blots were performed to validate these antibodies against canine samples. DNMT1 and IDH1 were both detectable in lysates of canine cells (Fig 1). For DNMT1, the bands were detected between 180 and 245 kDa, and the predicted molecular mass of DNMT1 is 183 kDa (Fig 1A). For IDH1, bands were detected at approximately 48 kDa, and the predicted molecular mass of IDH1 is 47 kDa (Fig 1B). 5MC and 5HMC are non-protein targets, molecularly identical across species, thus antibody validation was not required.

**Fig 2:**
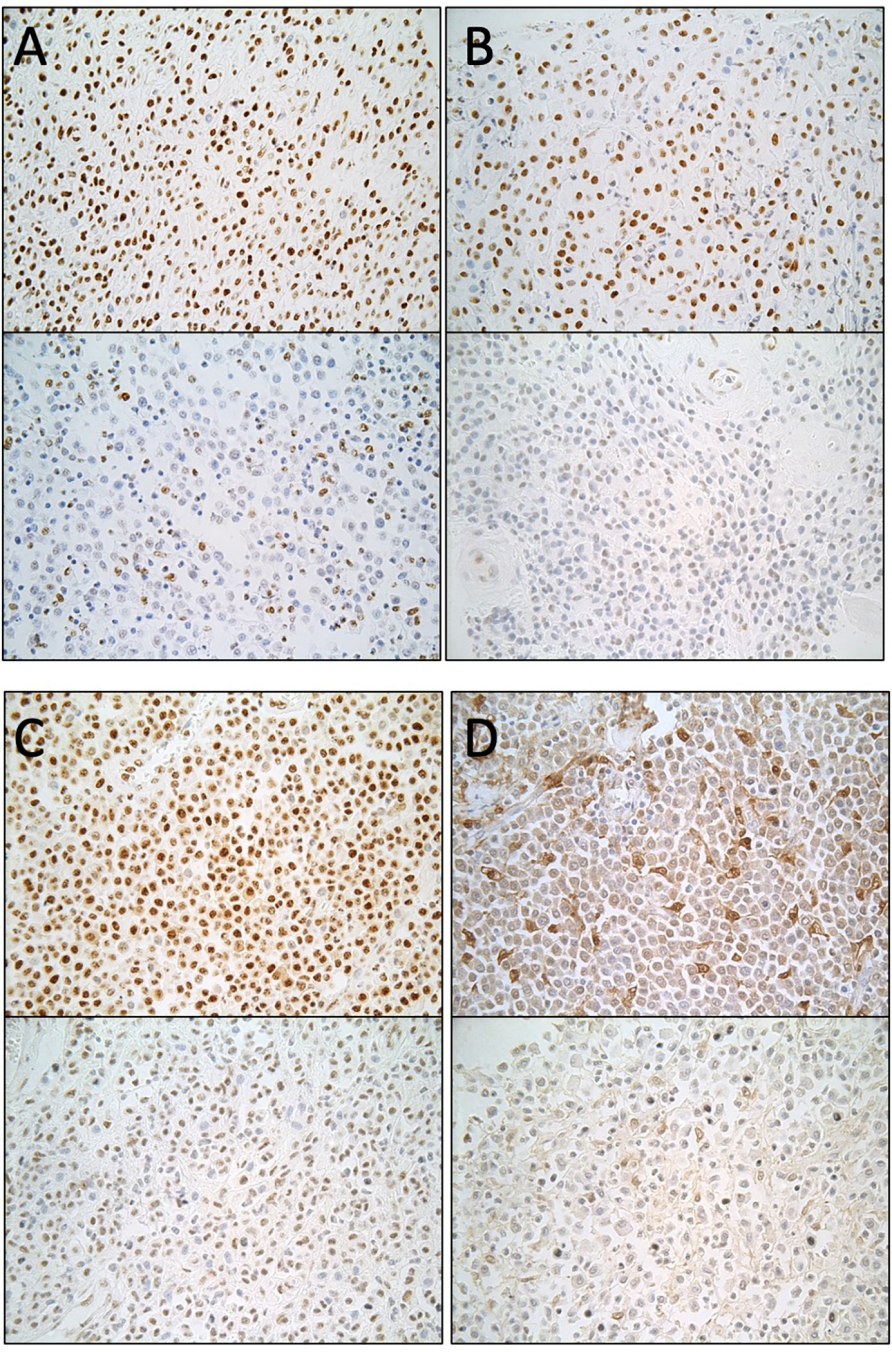
Brightfield images of TMA cores after immunohistochemistry and DAB detection. Top image in each pair shows an example of high H-score and bottom image of low H-score. **A**) 5-methylcytosine; **B**) 5- hydroxymethyl cytosine; **C**) DNMT1; **D**) IDH1. All images captured with 40X objective.

We then used immunohistochemistry to measure levels of 5MC, 5HMC, DNMT1, and IDH1 in our MCT TMAs and analyzed immunolabelling using QuPath as described above. Examples of cores with low and high H-scores for each marker are found in Fig 2.

After accounting for core loss during IHC, and elimination of cores that did not pass the quality control steps, cases with available disease-free interval (DFI) and overall survival (OS) data were used for outcome analysis. Table 1 shows the results of DFI analysis for dermal MCT. Low H-scores for 5MC and DNMT1 were associated with significantly longer DFI. High H-scores for IDH1 were associated with significantly longer DFI.

**Table 1:** Logrank analysis of disease free interval for all cases.

Parameter		N	Median Survival (Days)	Hazard Ratio (95% Confidence Interval)	P value
5MC	Low	62	undefined	0.4524 (0.2141–0.9561)	**0.0145**
	High	30	715		
5HMC	Low	40	undefined	0.5611 (0.2858–1.101)	0.0858
	High	49	1671		
DNMT1	Low	77	2079	0.4411 (0.2224–0.8750)	**0.0035**
	High	35	176		
IDH1	Low	33	1671	2.522 (1.127–5.644)	**0.0164**
	High	38	undefined		

Significant differences in DFI for subcutaneous cases are shown in Table 2. Low H-scores were associated with significantly longer OS for cases immunolabelled for 5MC and DNMT1. High H-scores were associated with significantly longer OS for cases immunolabelled with IDH1.

**Table 2:** Logrank analysis of survival time for all cases.

Parameter		N	Median Survival (Days)	Hazard Ratio (95% Confidence Interval)	P value
5MC	Low	77	1674	0.4585 (0.1801–0.7635)	**0.0070**
	High	38	577		
5HMC	Low	73	1671	0.5953 (0.2688–1.318)	0.1305
	High	16	752		
DNMT1	Low	64	1710	0.4987 (0.2717–0.9156)	**0.0123**
	High	44	577		
IDH1	Low	30	919	2.280 (1.088–4.774)	**0.0166**
	High	39	2054		

**Fig 3:**
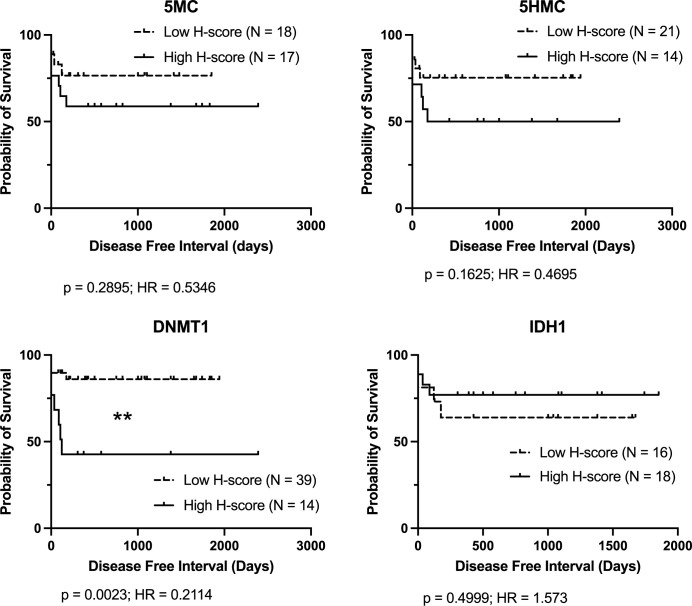
Kaplan-Meier survival curves for disease free interval (DFI) for dermal mast cell cancer cases stratified by immunolabelling H-score for each parameter. Logrank analysis indicates groups showing significant differences (p < 0.05). Cases lost to follow-up or still healthy at last follow-up are indicated by upward ticks on the curves, and were right-censored for analysis.

**Fig 4:**
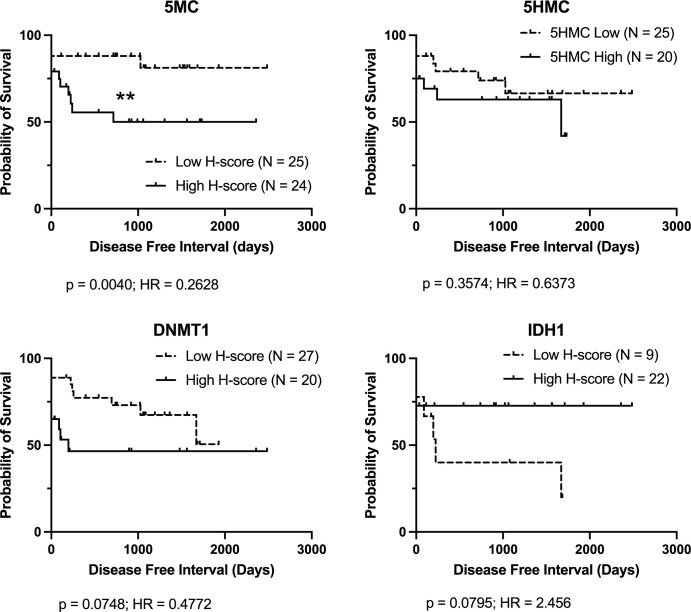
Kaplan-Meier survival curves for overall survival (DFI) for subcutaneous mast cell cancer cases stratified by immunolabelling H-score for each parameter. Logrank analysis indicates groups showing significant differences (p < 0.05). Cases lost to follow-up, still healthy at last follow-up, or that died of causes unrelated to MCT disease are indicated by upward ticks on the curves, and were right-censored for analysis.

Since there is evidence that subcutaneous MCT in general may be a less aggressive disease than dermal MCT, cases were also stratified by anatomical location and DFI outcome analysis was performed. For dermal cases, only low DNMT1 H-score was significantly associated with longer DFI (Fig 3). For subcutaneous cases, only low 5MC H-scores were associated with longer DFI (Fig 4).

Prognostication of canine MCTs based on grade can have variable outcome. Logrank analysis of DFI and OS was therefore conducted for cases stratified by Kiupel’s grading system grade. High grade cases with low H-scores for 5MC had significantly longer DFI (Table 3) and OS (Table 4). High grade cases with high DNMT1 H-scores showed significantly longer OS (Table 4), in contrast to what was found for all MCT cases (see Fig 4). The reasons for this difference are not clear, but could be related to DNMT1 mutation status, which was not evaluated in our study.

**Table 3:** Logrank analysis of disease-free interval for Kiupel’s grading system high grade cases only.

Parameter		N	Median Survival (Days)	Hazard Ratio (95% Confidence Interval)	P value
5MC	Low	78	undefined	0.4845 (0.2542–0.9234)	**0.0106**
	High	40	715		
5HMC	Low	40	undefined	0.5255 (0.1843–1.499)	0.2303
	High	36	undefined		
DNMT1	Low	59	undefined	0.4473 (0.1204–1.661)	0.1285
	High	17	undefined		
IDH1	Low	16	undefined	2.035 (0.4659–8.888)	0.2847
	High	32	undefined		

**Table 4:** Logrank analysis of overall survival for Kiupel’s grading system high grade cases only^¶^.

Parameter		N	Median Survival (Days)	Hazard Ratio (95% Confidence Interval)	P value
5MC	Low	62	undefined	0.2343 (0.0483–1.138)	**0.0109**
	High	19	1649		
5HMC	Low	52	undefined	0.4378 (0.1139–1.683)	0.1773
	High	23	1710		
DNMT1	Low	15	1649	4.982 (0.9463–26.24)	**0.0037**
	High	60	undefined		

^¶^ insufficient events in IDH1 groups for analysis

**Fig 5:**
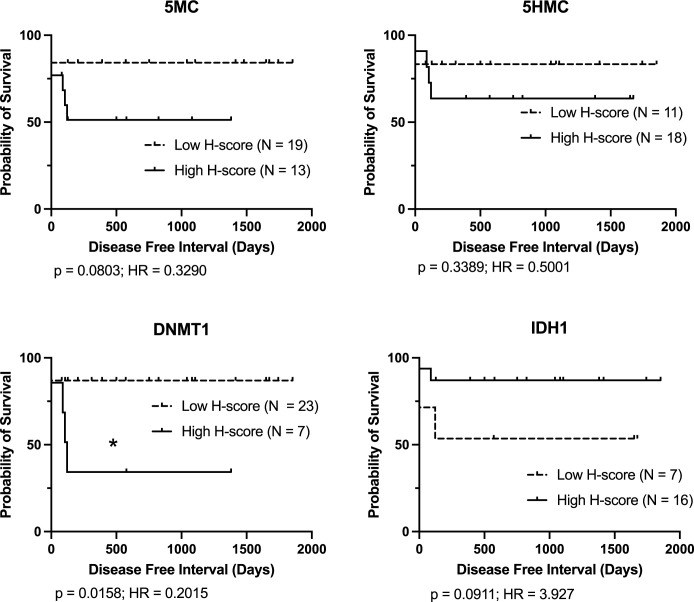
Kaplan-Meier survival curves for DFI for Patnaik’s grading system grade II cases. Logrank analysis indicates comparisons showing significant differences (p < 0.05). Cases lost to follow-up, still healthy at last follow-up, or that died of causes unrelated to MCT disease are indicated by upward ticks on the curves, and were right-censored for analysis.

**Fig 6:**
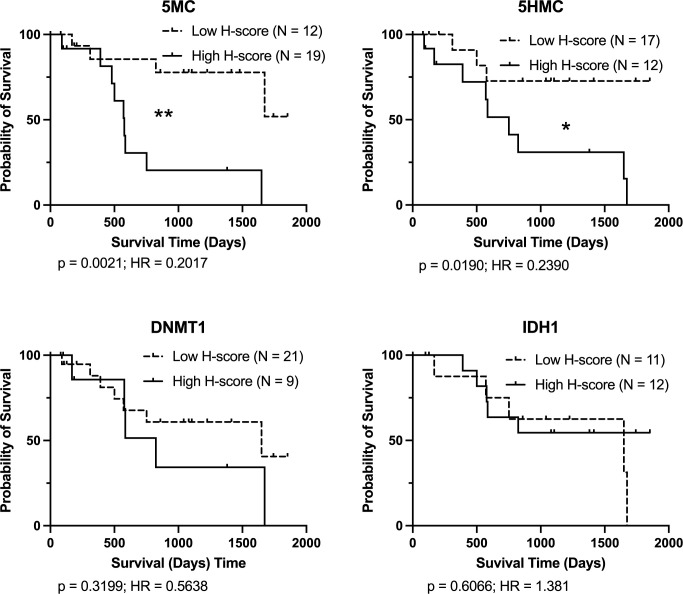
Kaplan-Meier survival curves for OS for Patnaik’s grading system grade II cases. Logrank analysis indicates comparisons showing significant differences (p < 0.05). Cases lost to follow-up, still healthy at last follow-up, or that died of causes unrelated to MCT disease are indicated by upward ticks on the curves, and were right-censored for analysis.

Cases graded with Patnaik’s grading system as grade II are the most difficult subset of MCT to determine the adequate therapy. We therefore also evaluated DFI and OS for these cases. Only low DNMT1 H-scores were significantly associated with better outcome as assessed by DFI (Fig 5). For OS of Patnaik grade II cases both low 5MC and low 5HMC H-scores were significantly associated with better outcome (Fig 6).

## Discussion

Our study found that high 5MC H-scores were significantly associated with poorer DFI and OS in canine MCTs when analyzing all cases. High 5MC H-scores also showed significance for poorer DFI and OS when stratifying the cases by Kiupel’s grading system high grade or Patnaik’s grading system grade II, and with poorer DFI when stratifying cases by subcutaneous location. A previous study found Kiupel’s grading system high grade canine MCT cases showed global hypomethylation, but that study did not evaluate case outcome [27]. In canine acute myeloid leukemia (AML), the DNA methylation profile was very heterogeneous compared to normal samples [28]. While neither hypermethylation nor hypomethylation alone was consistently associated with canine AML, there was both global hypermethylation in some cases and hypomethylation in others compared to normal samples [28]. In our study, high 5MC levels were associated with poorer outcome even when comparing dermal MCT to subcutaneous cases, which tend to be less aggressive and have better prognosis than similarly appearing grade 2 dermal MCT [17]. In human AML, a large component of chromatin differentially methylated regions were hypermethylated in high risk cytogenetic groups versus low and intermediate risk groups, hence prognosis could be determined from DNA methylation levels alone [29]. Being able to differentiate cases that are histologically nonaggressive but biologically aggressive using DNA methylation markers is important to ensure proper treatment options in canine MCT. In this study, high 5MC levels may be helpful in identifying more biologically aggressive cases and can thus aid in prognosis.

We also found that high DNMT1 H-scores were significantly associated with poorer DFI and OS in canine MCT when analyzing all cases. In addition, high DNMT1 H-scores were also significantly associated with worse DFI and OS for subcutaneous cases and with worse OS for Patnaik’s grading system grade II cases. However, low H-scores for DNMT1 were associated with worse OS for Kiupel’s grading system high grade cases. DNMT1 and DNMT3a were overexpressed in human AML compared to normal cells, and this upregulation was associated with hypermethylation of tumor suppressor genes associated with progression such as *p15* [30]. Overexpression of DNMTs is also reported in head and neck squamous cell carcinoma (HNSCC), where it was associated with higher pathological grade [31]. DNMTs are often mutated in various human hematologic malignancies including myeloid malignancies [3]. Mutations in DNMT1 and DNMT3a led to hypermethylation of promoter regions of several tumor suppressor genes including *p15* [32]. While mutational status was not evaluated in this present study, differences in canine MCT outcome were associated with DNMT1 protein expression.

We found that low IDH1 H-scores were significantly associated with poorer DFI and OS in canine MCTs when analyzing all cases. Normally, IDH1 and/or 2 catalyze the oxidative decarboxylation of isocitrate to α-ketoglutarate in the tricarboxylic acid cycle and α-ketoglutarate along with Fe(II) is an important cofactor in normal TET2 enzyme function, associated with DNA demethylation [6]. When mutated, IDH1/2 often further convert α-ketoglutarate into D-2HG which acts as an agonist for TET2 and along with reducing the supply of α-ketoglutarate, inhibits normal TET2 function [6, 10]. Human AML cells harbouring IDH1 and/or 2 mutations had increased global methylation status compared to wildtype CD34+ bone marrow cells, possibly due to increased D-2HG levels in mutant cells [10]. IDH mutated AML cells actively inhibited the functions of TET2 enzymes through these increased levels of D-2HG, leading to an increase in global 5MC levels [10]. AML patient samples with IDH1/2 mutations had significant DNA hypermethylation compared to normal bone marrow samples taken from healthy patients. That study reported 154 hypermethylated genes, of which a majority had more than 2-fold decreased expression [10]. IDH mutations in murine models of glioma led to disease progression with the associated upregulated D-2HG led to global hypermethylation, establishing a phenotype in gliomas called glioma CpG island methylator phenotype [33]. Human AML cases with both TET2 mutations and IDH mutations showed similar hypermethylated patterns, however IDH mutations were associated with a greater amount of promoter hypermethylation and global hypermethylation [10]. Collectively, this supports the findings of our study that dysregulation of IDHs could be prognostic for canine MCT.

The antibodies for enzymes used for this study were not developed using canine peptides, thus validation was necessary to confirm detection of the relevant proteins in canine cells. Our original study plan included evaluating DNMT3a, TET1, TET2 and IDH2 in addition to the reported biomarkers. However, we were unsuccessful in validating these markers with the antibodies available, which is a limitation of our study. The benefit of using TMAs is that a large patient cohort can be analyzed simultaneously with identical immunolabelling conditions, thus avoiding technical variability in the detection of target antigens in the tissues [34, 35]. However, we encountered more core loss in the IDH1 immunolabelled TMAs than in the TMA sections used to detect 5MC, 5HMC, and DMNT1. Since there were up to three cores taken from many cases, the loss of cores did not necessarily always lead to the loss of a case from further analysis, but we cannot discount the possibility that this loss potentially affected our findings [34, 35]. Another limitation is the retrospective nature of our analysis, as there can be bias in information available due to uncollected data that can occur in retrospective studies [36].

## Conclusions

We evaluated markers associated with altered DNA methylation and found a statistically significant difference between the DFI and OS of canine MCTs immunolabelled for 5MC, DNMT1, and IDH1. High levels of 5MC and DNMT1 were also associated with differential DFI and OS, in all cases and in some subcategories (*e*.*g*. anatomic location; Kiupel’s grading system grade). Further studies are required to determine if these parameters can potentially be used to stratify canine MCT patients for treatment with DNA methylation inhibitors.

## Supporting information

S1 Fig Uncropped, unadjusted images of western blots. (PDF)

S2 Fig Brightfield images of TMA cores showing positive and negative immunolabelling. For all image pairs, positive immunolabelling is on the left and negative immunolabelling is on the right. **A)** 5-methylcytosine; **B)** 5-hydroxymethylcytosine; **C)** DNMT1; **E)** IDH1. All images captured with 20X objective.
(PDF)

S3 Fig QuPath parameters. Settings and set-up parameters for detection and quantification of immunolabelling for each parameter using QuPath software.
(PDF)
