# Supplementary material for: Prognostic value of epigenetic markers for canine mast cell cancer
Source: PLoS One. 2023 Mar 30;18(3):e0283616. doi: 10.1371/journal.pone.0283616 (PMC10062589; doi:10.1371/journal.pone.0283616)
Supplement: S1 Fig — (PDF) [file pone.0283616.s001.pdf]

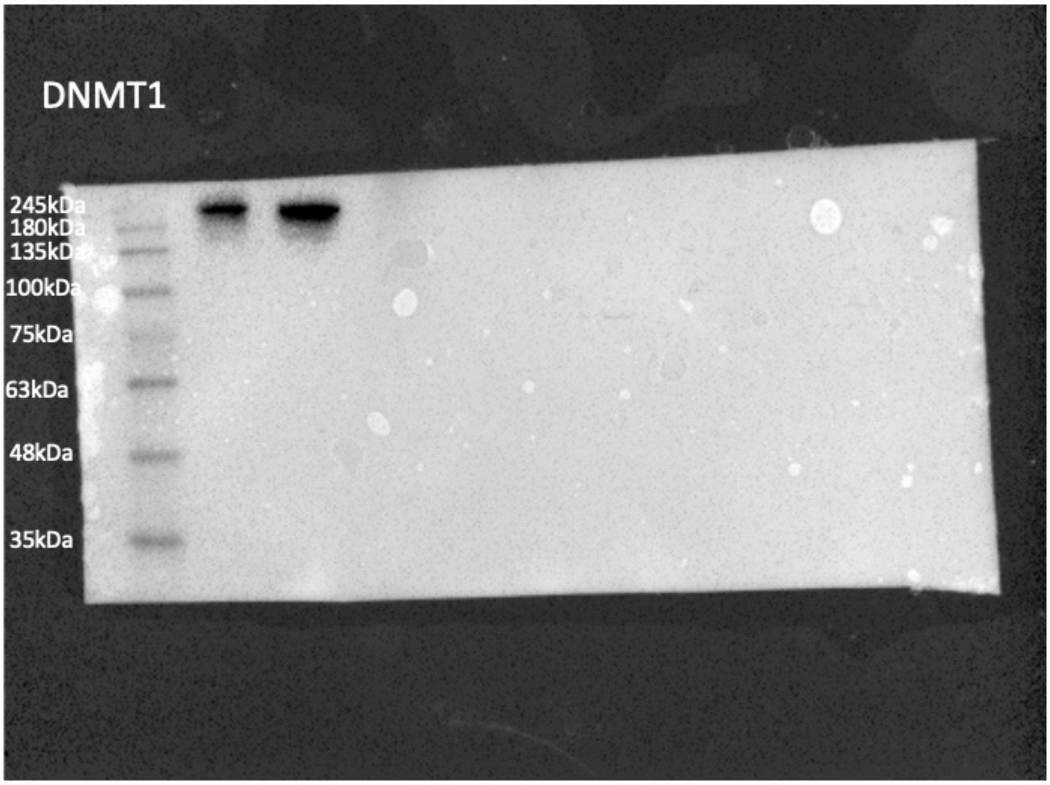

lane 1: Molecular mass markers; lane 2: MCT1 canine mast cell cancer cell line; lane 3: MCT2 canine mast cell cancer cell line

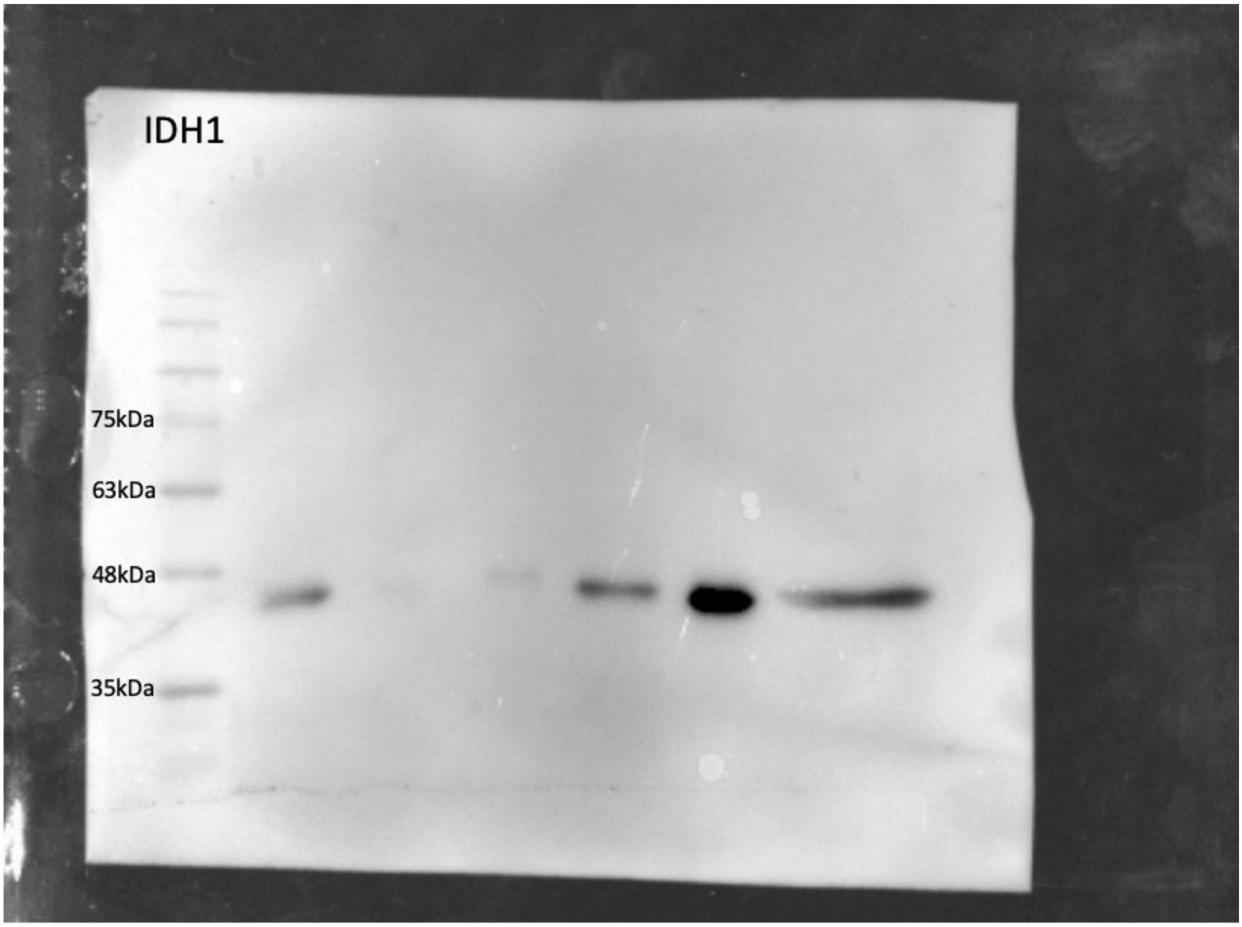

lane 1: Molecular mass markers; lane 2: MCT1 canine mast cell cancer cell line; lane 3: MCT2 canine mast cell cancer cell line; lane 4: CBCL1 canine lymphoma cell line; lane 5: MDCK cell line; lane 6: normal human liver lysate; lane 7: HeLa cell lysate
