# Supplementary figures and images for: Prognostic value of epigenetic markers for canine mast cell cancer
Source: PLoS One. 2023 Mar 30;18(3):e0283616. doi: 10.1371/journal.pone.0283616 (PMC10062589; doi:10.1371/journal.pone.0283616)

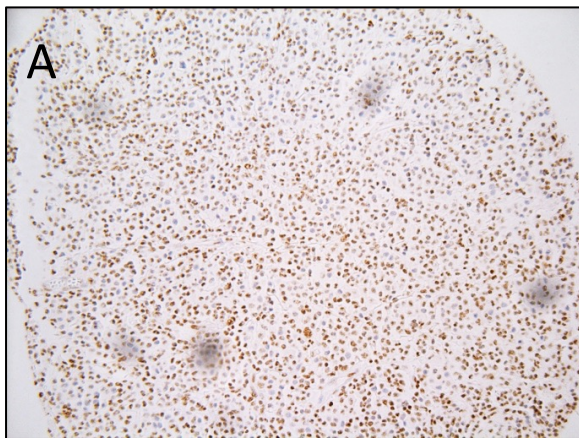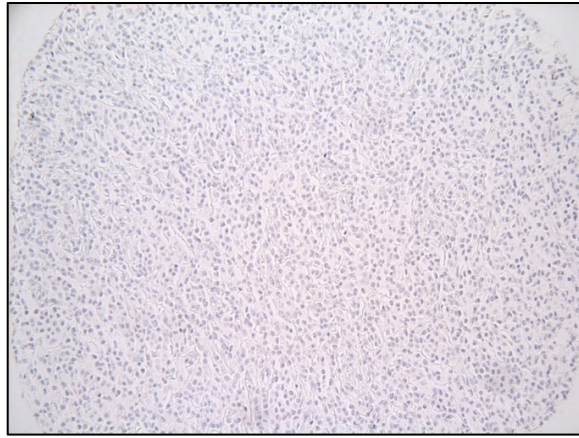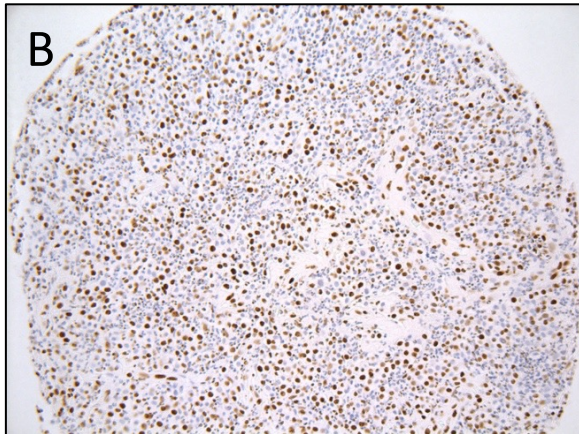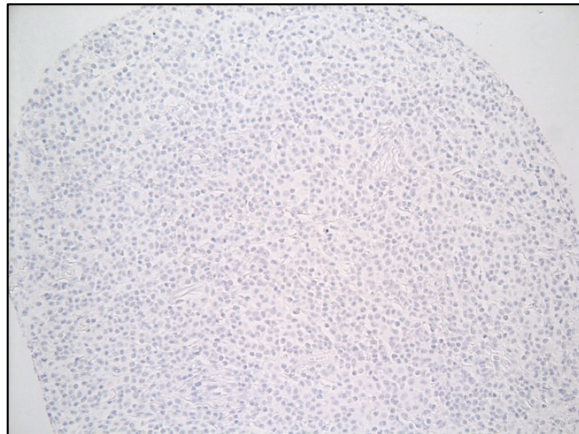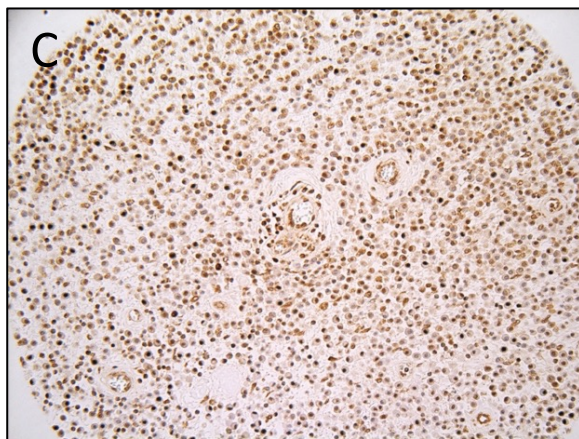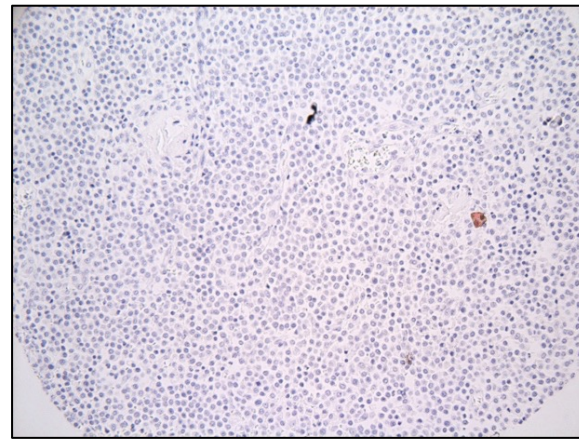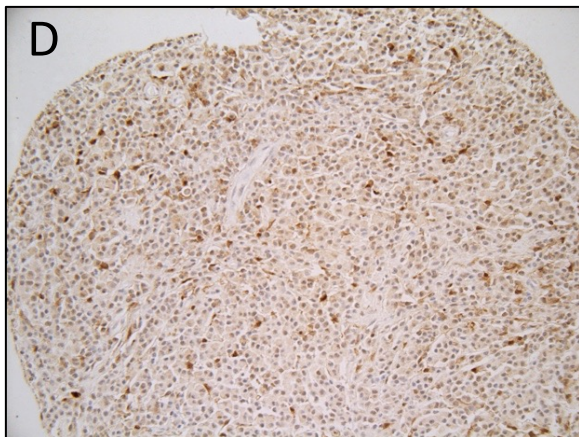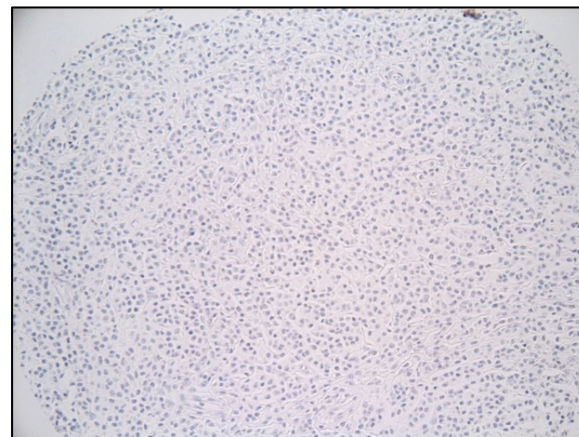

Supplement: S2 Fig — For all image pairs, positive immunolabelling is on the left and negative immunolabelling is on the right. A) 5-methylcytosine; B) 5-hydroxymethylcytosine; C) DNMT1; E) IDH1. All images captured with 20X objective. (PDF) [file pone.0283616.s002.pdf]
