# Supplementary material for: Prognostic value of epigenetic markers for canine mast cell cancer
Source: PLoS One. 2023 Mar 30;18(3):e0283616. doi: 10.1371/journal.pone.0283616 (PMC10062589; doi:10.1371/journal.pone.0283616)
Supplement: S3 Fig — Settings and set-up parameters for detection and quantification of immunolabelling for each parameter using QuPath software. (PDF) [file pone.0283616.s003.pdf]

5MC QuPath parameters

Positive cell detection

Parameter	Value	Unit
**Setup parameters**		
Choose detection image	Optical density sum	
Requested pixel size	0	µm
**Nucleus parameters**		
Background radius	8	µm
Median filter radius	0	µm
Sigma	1.5	µm
Minimum area	8	µm^2
Maximum area	100	µm^2
**Intensity parameters**		
Threshold	0.1	
Max background intensity	2	
Split by shape	☑	
Exclude DAB (membrane staining)	☑	
**Cell parameters**		
Cell expansion	3 µm	
Include cell nucleus	☑	
**General parameters**		
Smooth boundaries	☑	
Make measurements	☑	
**Intensity threshold parameters**		
Score compartment	Nucleus: DAB OD mean	
Threshold 1+	0.11	
Threshold 2+	0.24	
Threshold 3+	0.38	
Single threshold	☐	

Run

5HMC QuPath parameters

Positive cell detection

Parameter	Value	Unit
**Setup parameters**		
Choose detection image	Optical density sum	
Requested pixel size	0.5	µm
**Nucleus parameters**		
Background radius	8	µm
Median filter radius	0	µm
Sigma	1.5	µm
Minimum area	8	µm^2
Maximum area	100	µm^2
**Intensity parameters**		
Threshold	0.1	
Max background intensity	2	
Split by shape	☑	
Exclude DAB (membrane staining)	☑	
**Cell parameters**		
Cell expansion	3 µm	
Include cell nucleus	☑	
**General parameters**		
Smooth boundaries	☑	
Make measurements	☑	
**Intensity threshold parameters**		
Score compartment	Nucleus: DAB OD mean	
Threshold 1+	0.13	
Threshold 2+	0.23	
Threshold 3+	0.4	
Single threshold	☐	

Run

DNMT1 QuPath parameters

Positive cell detection

Parameter	Value	Unit
**Setup parameters**		
Choose detection image	Hematoxylin OD	
Requested pixel size	0.5	µm
**Nucleus parameters**		
Background radius	8	µm
Median filter radius	0	µm
Sigma	1.4	µm
Minimum area	8	µm^2
Maximum area	100	µm^2
**Intensity parameters**		
Threshold	0.07	
Max background intensity	2	
Split by shape	☑	
Exclude DAB (membrane staining)	☐	
**Cell parameters**		
Cell expansion	2.5 µm	
Include cell nucleus	☑	
**General parameters**		
Smooth boundaries	☑	
Make measurements	☑	
**Intensity threshold parameters**		
Score compartment	Nucleus: DAB OD mean	
Threshold 1+	0.18	
Threshold 2+	0.36	
Threshold 3+	0.57	
Single threshold	☐	

Run

IDH1 QuPath parameters

Positive cell detection

Parameter	Value	Unit
**Setup parameters**		
Choose detection image	Hematoxylin OD	
Requested pixel size	0.5	µm
**Nucleus parameters**		
Background radius	8	µm
Median filter radius	0	µm
Sigma	1.6	µm
Minimum area	8	µm^2
Maximum area	100	µm^2
**Intensity parameters**		
Threshold	0.08	
Max background intensity	2	
Split by shape	☑	
Exclude DAB (membrane staining)	☐	
**Cell parameters**		
Cell expansion	1.5 µm	
Include cell nucleus	☑	
**General parameters**		
Smooth boundaries	☑	
Make measurements	☑	
**Intensity threshold parameters**		
Score compartment	Cell: DAB OD mean	
Threshold 1+	0.22	
Threshold 2+	0.35	
Threshold 3+	0.5	
Single threshold	☐	

Run
